# Die-Attach Structure of Silicon-on-Glass MEMS Devices Considering Asymmetric Packaging Stress and Thermal Stress

**DOI:** 10.3390/s19183979

**Published:** 2019-09-14

**Authors:** Jun Eon An, Usung Park, Dong Geon Jung, Chihyun Park, Seong Ho Kong

**Affiliations:** 1 School of Electronics Engineering, College of IT Engineering, Kyungpook National University, Daegu 41566, Korea; 2 The 3rd R&D Institute—4th Directorate, Agency for Defense Development, Daejeon 34186, Korea; 3 Micro-infinity Co. Ltd., Suwon 16229, Korea

**Keywords:** die attach, thermal stress, microelectromechanical system (MEMS), asymmetric packaging stress, differential resonant accelerometer (DRA), silicon-on-glass process, glass-on-silicon wafer

## Abstract

Die attach is a typical process that induces thermal stress in the fabrication of microelectromechanical system (MEMS) devices. One solution to this problem is attaching a portion of the die to the package. In such partial die bonding, the lack of control over the spreading of the adhesive can cause non-uniform attachment. In this case, asymmetric packaging stress could be generated and transferred to the die. The performance of MEMS devices, which employ the differential outputs of the sensing elements, is directly affected by the asymmetric packaging stress. In this paper, we proposed a die-attach structure with a pillar to reduce the asymmetric packaging stress and the changes in packaging stress due to changes in the device temperature. To verify the proposed structure, we fabricated four types of differential resonant accelerometers (DRA) with the silicon-on-glass process. We confirmed experimentally that the pillar can control the spreading of the adhesive and that the asymmetric packaging stress is considerably reduced. The simulation and experimental results indicated that the DRAs manufactured using glass-on-silicon wafers as handle substrates instead of conventional glass wafers have a structure that compensates for the thermal stress.

## 1. Introduction

**Figure 1:**
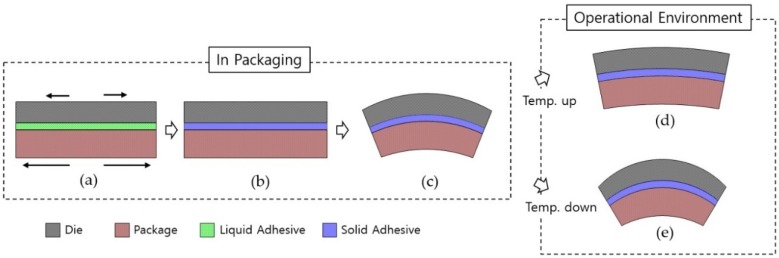
Change in Die Warpage: (**a**) T < adhesive curing temp.; (**b**) T = adhesive curing temp.; (**c**) cooling to room temp.; (**d**) room temp. < T < adhesive curing temp.; (**e**) T < room temp.

The fabrication of microelectromechanical system (MEMS) devices commonly involves bonding two materials having different coefficients of thermal expansion (CTEs). Given the high bonding temperatures involved, MEMS devices are adversely affected by thermal stress. Die attach is the most important step in the packaging of high-precision MEMS sensors, and this bonding process typically induces thermal stress [1,2,3]. Generally, the packaging material of a MEMS sensor has a greater CTE than that of the die material, such as silicon and glass. Hence, during the packaging process, the package expands more than the die without any restriction until the curing temperature of the adhesive is reached (Figure 1a). The package and die expand to different proportions and are then fixed to each other with a solidified adhesive at the curing temperature (Figure 1b). As the fabricated device cools down to room temperature, the thermal stresses induced by the difference in the shrinkage ratios of the package and the die cause the bonded assembly to deform into a concave downward shape (Figure 1c). In an operational environment, this distortion of the die, or die warpage or packaging stress, decreases as the temperature increases and increases as the temperature decreases (Figure 1d,e).

The packaging stress causes structural deformation of devices, such as buckling of long beam elements [4]. Changes in the packaging stress due to temperature induce performance drift [5]. Therefore, the packaging stress and the changes in the packaging stress should be suppressed. To these ends, conventional approaches employ mechanical or thermal isolators [6,7,8,9,10,11], low-stress adhesives [12,13], and calibration [14,15,16]. An isolator with a compliant beam element can decouple a structure from packaging stress. The application of this approach may be limited by the structural design of the device, or it may degrade sensor performance by generating mechanical resonance [17]. The use of a micro oven as a thermal isolator increases the system complexity and power consumption. A low-stress adhesive, which has a low modulus and can be cured at low temperatures, can reduce the packaging stress. Typically, polymer-based materials meet these requirements. However, because of poor bonding strength, they are unsuitable for applications requiring high reliability and are used primarily in applications requiring low performance [1,18]. Calibration methods using external temperature sensors have limitations associated with thermal delays and temperature hysteresis [15]. 

Another approach to this problem is to simply reduce the bonding area of the die and the package [19,20,21]. Attaching the die to the package with a few adhesive dots can reduce the packaging stress and variations in the packaging stress. Multiple adhesive dots can be used to achieve a strong bond. A four-dots die attach is used in the Motorola packaging factory [22].

Time-pressure dispensing is the most commonly used method to dispense very small volumes, such as adhesive dots. However, with this method, it is difficult to precisely control the amount of adhesive applied [23,24,25]. In a multi-dot die attach, such as a four-dots die attach, when the attachment states of the adhesive dots are different, asymmetric packaging stresses are generated and transferred to the die. The performance of a high-precision sensor that uses the differential outputs of stress-sensing elements, such as a differential resonant accelerometer (DRA), is directly affected by the asymmetric packaging stresses [26]. 

In this paper, we proposed a die-attach structure based on a pillar structure to reduce the asymmetric packaging stress and the changes in the packaging stress due to temperature. To verify the proposed structure, we fabricated four types of DRAs using two types of handle substrates (glass-on-silicon (GOS) wafers and glass wafers) and two die-attach schemes (pillar and four-dots). Stress evaluation was performed by measuring the out-of-plane displacement of the DRA structure with an optical profiler. We evaluated asymmetric packaging stresses by measuring the deformation of the DRA proof masses before and after packaging. The asymmetric packaging stresses were compared with the resonant frequency differences between the two sensing elements in the DRA. The changes in the packaging stress due to temperature were measured based on the changes in die warpage. In addition, the spreading shape of the adhesive according to the die attach was examined, and the processability of pillar formation was compared according to the type of handle substrate. For the change in die warpage due to temperature, the simulated and experimental results were compared. The thermal-stress-compensation mechanism involving the bending of two bonding interfaces in opposite directions due to temperature change was also discussed.

## 2. Structure of Test Devices

### 2.1. GOS Wafer

The GOS wafer was prepared through anodic bonding of a glass wafer and a silicon wafer. To achieve a certain final thickness of the glass layer, the bonded wafer was thinned by grinding and chemical mechanical planarization (CMP). The GOS wafer has been used as a basic substrate for MEMS applications, such as the base substrate in a MEMS cap wafer. Researchers can fabricate and employ GOS wafers using process equipment. However, it is more convenient to use commercial products sold by professional manufacturers, such as regular wafers. In this study, we used a 6-in. GOS wafer manufactured by Plan Optik (Elsoff, Germany). The thicknesses of the glass layer and the silicon layer were 23 μm and 680 μm, respectively.

### 2.2. DRA

**Figure 2:**
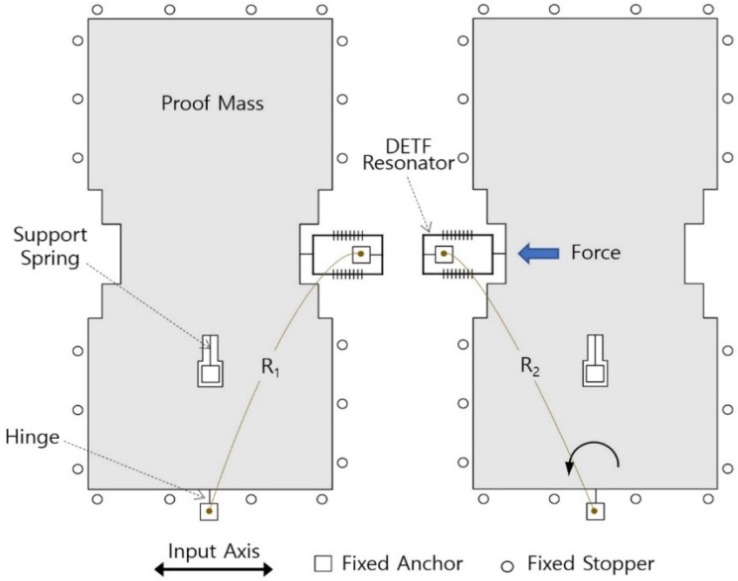
Schematic Diagram of the differential resonant accelerometers (DRA). DETF: double-ended tuning fork.

The test DRA used in this study is shown in Figure 2. When acceleration along the input axis is applied to the DRA, the proof mass generates axial force on the double-ended tuning fork (DETF) resonator. Due to the push-pull structure, the left and right DETF resonators are forced in different direction. That is, when one resonator is subjected to tension and its resonant frequency increases, the other one is compressed and its resonant frequency decreases. The applied acceleration is measured by detecting the difference between the resonant frequencies of the two DETF resonators. Because two identical structures are arranged symmetrically, this differential design doubles the sensitivity of the accelerometer and eliminates common-mode errors, such as temperature and stress [27,28]. 

Given no acceleration input, the bias of the DRA is defined as the output frequency difference in the two DETF resonators. To improve the bias stability of the accelerometer, bias is reduced as much as possible, depending on how close the resonant frequencies of the two DETF resonators are to each other. The primary reason for the difference in the resonant frequencies of the two DETF resonators is the mismatches in the beam width and the residual stress [29]. By measuring the electrical resistances (R_1_ and R_2_ in Figure 2) between the hinge and the DETF resonator, it is possible to choose dies with DETF resonators whose resonant frequencies are similar. This method takes advantage of the tendency that the electrical resistances R_1_, R_2_ are primarily determined by the beam width of the DETF resonator. In our manufacturing facility, this method is required to screen the DRA dies before packaging. The test DRA dies used in this study were those with resonant frequency differences between the two DETF resonators of less than 60 Hz. 

On the other hand, unlike the typical DRAs, the left and right proof masses of the test DRA were completely separated to prevent the lock-in phenomenon [30]. By restricting the excessive motion of the proof mass, the cylindrical stopper around the proof mass would prevent hinge breakage.

### 2.3. Types of Test Devices

**Figure 3:**
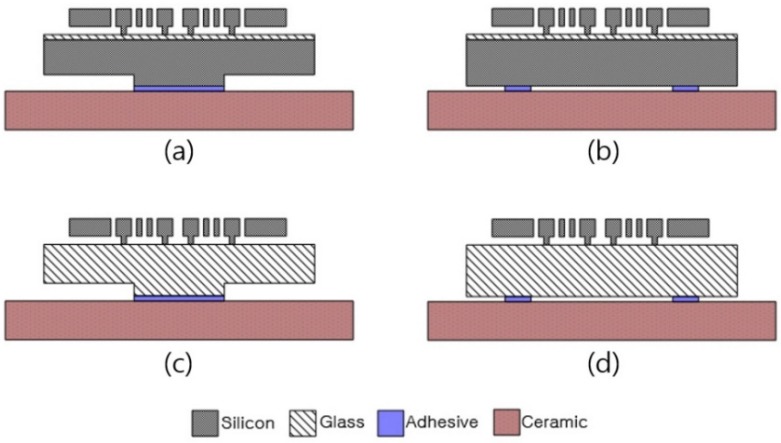
Cross-sectional Views of the Four Types of DRAs (not to scale): (**a**) Type I-A; (**b**) Type I-B; (**c**) Type II-A; and (**d**) Type II-B.

**Figure 4:**
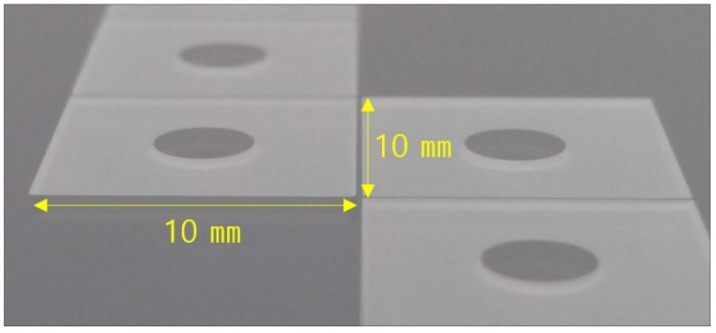
Optical Image of Glass Pillars.

Figure 3 shows the four types of test devices used in this study. All the devices had the same silicon device structure. The model using the GOS wafers as the handle substrate was defined as Type I and the model using the glass wafers was defined as Type II. Type I and Type II were subdivided into A and B according to the die-attach scheme (Figure 3, Table 1). The pillar die attach used a pillar, a short cylindrical structure formed on the bottom of the die (Figure 4). The four-dots die attach used a flat bottom surface. The diameter and height of the pillars were 3400 μm and 70 μm, respectively. The pillars occupied 9% of the die area (10 × 10 mm^2^).

## 3. Chip Fabrication and Die Attach

### 3.1. Chip Fabrication

The silicon-on-glass (SOG) process can easily increase the thickness of the active silicon layer. Because the sensitivity of an accelerometer is proportional to the mass of the proof mass, SOG process is effective for manufacturing highly sensitive MEMS accelerometers. The test DRA used in this study was fabricated using the SOG process. 

A 500 μm thick silicon wafer was used to fabricate the active silicon layer. A GOS wafer with a thickness of 703 (23 + 680) μm and a Pyrex 7740 glass wafer with a thickness of 680 μm were used as the handle substrates for the Type I and Type II devices, respectively. 

#### 3.1.1. Type I Device

**Figure 5:**
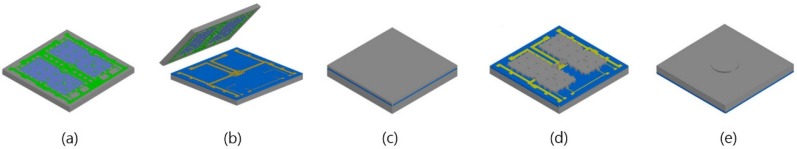
Chip Fabrication Process for Type I Device: (**a**) anchor formation; (**b**) electrode patterning and anodic bonding; (**c**) silicon thinning by grinding and chemical mechanical planarization (CMP); (**d**) detailed structure formation; and (**e**) pillar formation by deep reactive ion etching (DRIE).

The chip manufacturing process is shown in Figure 5 and its steps are described as follows.

1. Recesses were etched into a silicon wafer using dry etching. An anti-footing Al layer was deposited and patterned to prevent footing in the deep reactive ion etching (DRIE) process (Figure 5a).
2. Electrodes were formed on the glass layer of the GOS wafer. Anodic bonding was performed below 400 °C to prevent the anti-footing Al layer from diffusing into the silicon layer (Figure 5b).
3. The thickness of the silicon device layer was thinned to 80 μm by lapping and CMP (Figure 5c).
4. The silicon device structure was formed using the DRIE process. The remaining silicon was removed except for the silicon device structure (Figure 5d).
5. The Type I-A device involved a pillar-formation step. The pillar was formed using the DRIE process (Figure 5e).

#### 3.1.2. Type II Device

**Figure 6:**
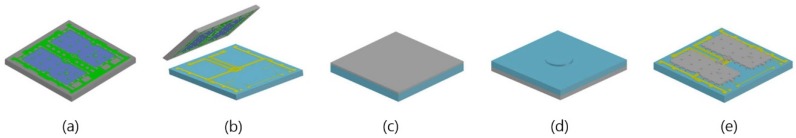
Chip Fabrication Process for Type II: (**a**) anchor formation; (**b**) electrode patterning and anodic bonding; (**c**) silicon thinning by grinding and CMP; (**d**) pillar formation by powder blasting; and (**e**) detailed structure formation.

The chip manufacturing process for Type II was the same as that for Type I except for a pillar-formation step (Figure 6). In the pillar-formation step, a dry film photoresist was patterned on the bottom of the Type II-A device. The pillar was formed using powder blasting. During this process, the thinned front silicon layer was protected by photoresist (Figure 6d).

### 3.2. Packaging Procedure

The die adhesive TB3303N, manufactured by Threebond (Tokyo, Japan), is a silicon-based conductive adhesive containing an Ag filler. It has a low viscosity (41 pa·s @ RT) that is beneficial for dispensing it finely. 

**Figure 7:**
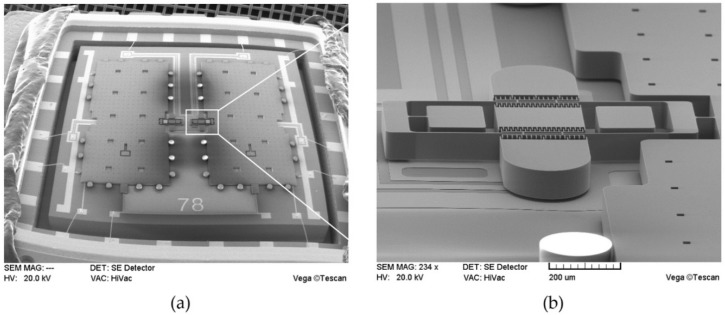
SEM (scanning electron microscopy) Image: (**a**) packaged DRA and (**b**) close up of the DETF resonator.

The die attach process proceeded in two steps. In the first step, the adhesive was applied over the ceramic package according to the time-pressure dispensing method. For dot dispensing, the pneumatic controller was set to dispense two times at 0.4 MPa in 0.2 s. A dispensing robot precisely controlled the dot-dispensing position. In the second step, the die was placed on the adhesive using a die bonder and a bond force was applied. The packaged die was then transferred to a hot plate to cure the adhesive for 1 h at 180 °C. Subsequent packaging processes included wire bonding, degassing, vacuum sealing, and getter activation. The final thickness of the adhesive formed between the die and the package was approximately 35 μm. The packaged test DRA is shown in Figure 7. 

## 4. Results and Discussion

### 4.1. Asymmetric Packaging Stress

Because the proof mass is supported by three beam elements (DETF resonator, support spring, and hinge), the deformation of the proof masses could be ascribed to a change in the stress between the beam elements. This implies a change in the stress state of the DETF resonators. When a similar-sized deformation occurs in both proof masses, both DETF resonators undergo a similar stress change. However, if the deformations of the left and right proof masses occur at different ratios, both DETF resonators would undergo different stress changes. In this case, the differential output of the DRA (bias) would increase, even if there is no acceleration input. Therefore, for the sensors with differential output, such as the DRAs, asymmetric packaging is an important issue that affects the DRA bias.

**Figure 8:**
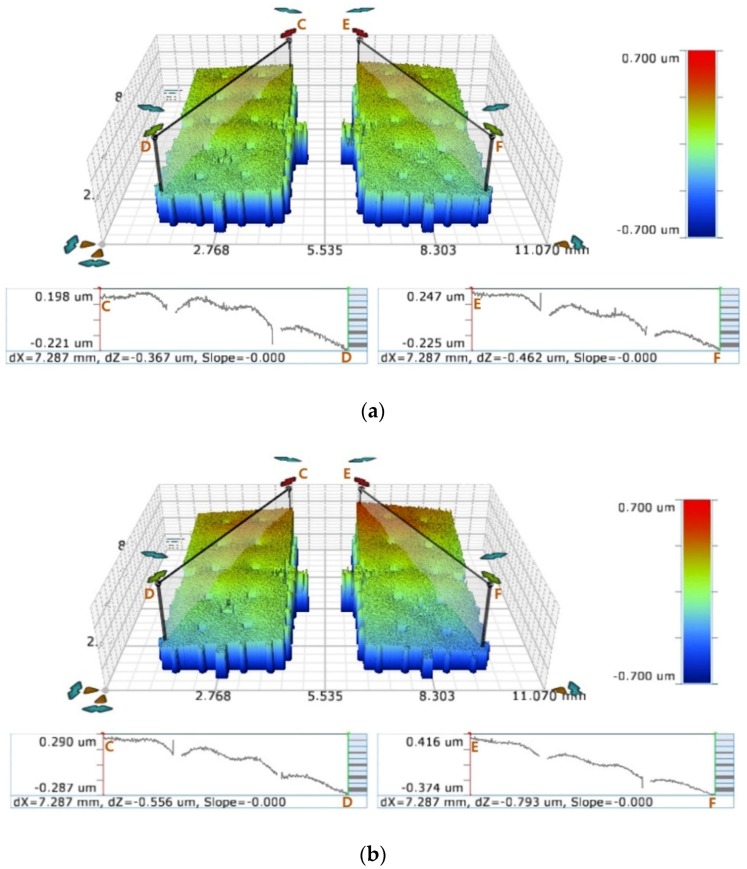
Deformation of proof masses in Type I-B device: (**a**) before packaging and (**b**) after packaging.

In this study, we evaluated the asymmetric packaging stress as the ratio of the slope changes of both proof masses and then compared them with the resonant frequency difference of the two DETF resonators. First, we measured the out-of-plane displacement of the DRAs before and after the packaging process using an optical profiler. The equipment was comprised of a white-light interferometric microscope with a vertical resolution of 0.03 nm modified to accommodate the heating stage. We then measured the height difference dZ between the two opposing edges of the proof masses before packaging and then obtained the change in dZ after packaging (Figure 8). 

Table 2 shows the deformation ratios of both proof masses in the test DRAs before and after packaging. As the deformation ratio increases beyond 1, the asymmetric packaging stress increases. The asymmetric packaging stress was considerably greater in the four-dots die attach than in the pillar die attach.

Table 3 shows the resonant frequency difference of the two DETF resonators in the vacuum-sealed test DRAs (a digital phase-locked loop was used for resonant frequency measurements). As described in Section 2.2, the resonant frequency differences of the test DRA dies selected for the study were less than 60 Hz, but the differential outputs of the test DRAs with four-dots die attach increased significantly compared to that of the pillar die attach. This result demonstrated that asymmetric packaging stress caused the increase in the output frequency difference of the test DRAs.

### 4.2. Non-Uniform Attachment in the Four-Dots Die Attach

The asymmetric packaging stress is induced by various error factors in the die-attach process. The most common error factor is the difference in the attachment area. It is difficult to make the bonding areas of each of the adhesive dots uniform using the time-pressure dispensing. This is primarily due to the fact that the amounts of dispensed adhesive dots are not the same. According to another study that compared the amounts of dispensed adhesive dots, the time-pressure dispensing showed a large variation of 9.7–36.5% in the same process [25]. This confirmed that it is difficult to control the bonding areas of each adhesive dots as well as the total bonding area of each adhesive using the time-pressure dispensing.

**Figure 9:**
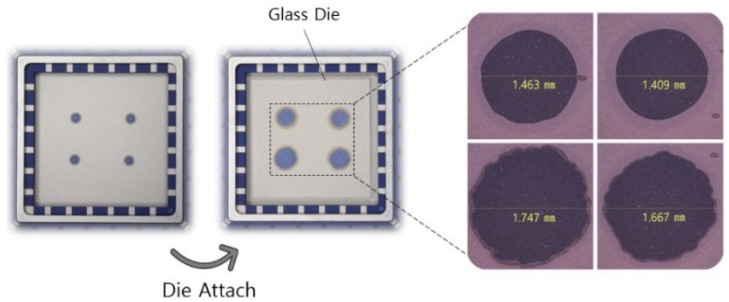
Die attach pattern and spreading shape of the die adhesive in the four-dots die attach.

To observe the adhesive spread shape in the four-dots die attach, the silicon device layer of the Type II-B device was removed with tetramethyl ammonium hydroxide (TMAH) and the die attach process performed. As shown in Figure 9, and as expected, the bonding areas of each of the adhesive dots were not uniform. 

### 4.3. Fillet Effect in the Pillar Die Attach

**Figure 10:**
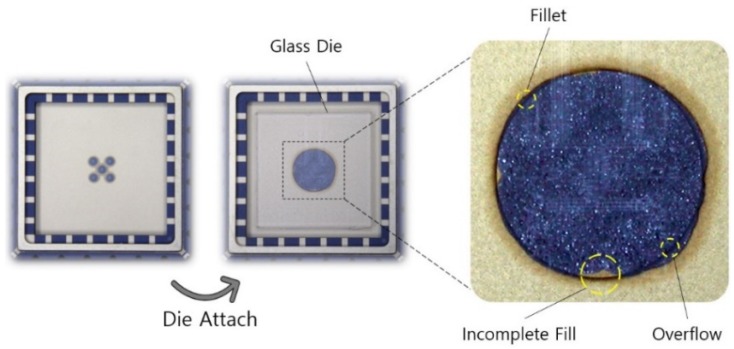
Die-attach pattern and spreading shape of the die adhesive in the pillar die attach.

**Figure 11:**
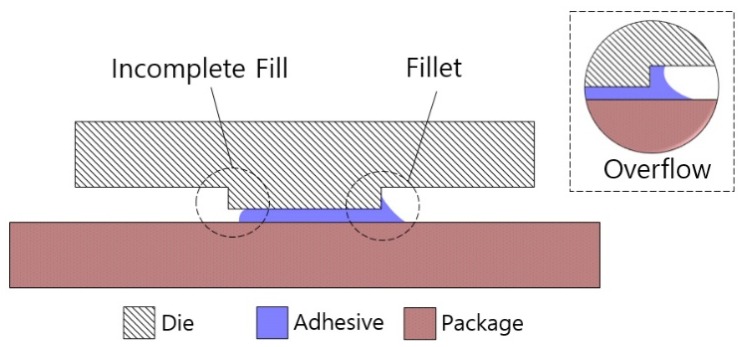
Cross-sectional view of the pillar die attach (not to scale).

The spreading shape of the adhesive in the pillar die attach was confirmed using the method described in Section 4.2. In the pillar die attach, the fillet, incomplete fill, and overflow displayed in Figure 10 and Figure 11 were observed at the edge of the pillar. The fillet acted as a reservoir to accommodate for variations such as the amount of adhesive, location of adhesive, and misalignment of die and substrate. Incomplete fill and overflow (bad fillets) are two important defects that occur in both the typical die attach process and the pillar die attach. The solution to this problem is optimizing the process parameters, such as the amount of adhesive, bonding force, location of the adhesive, and pillar height. As shown in Figure 10, the incomplete fill area and the overflow area accounted for only 0.7% and 1.1% of the pillar area, respectively. As a result, the pillar die attach, which employed the fillet effect, could easily control the adhesive spreading area and made the attachment area symmetric and uniform. 

In this study, on the other hand, we experimentally determined the average value of the bonding area of the four-dots die attach and determined the diameter of the pillar. In this way, the four-dots die attach and pillar die attach were intended to have the same bonding area. However, due to the variation of the time-pressure dispensing, the bonding area of the four-dots die attach and pillar die attach could not be the same.

### 4.4. Comparison of Pillar Fbrication Processability and Yield

**Figure 12:**
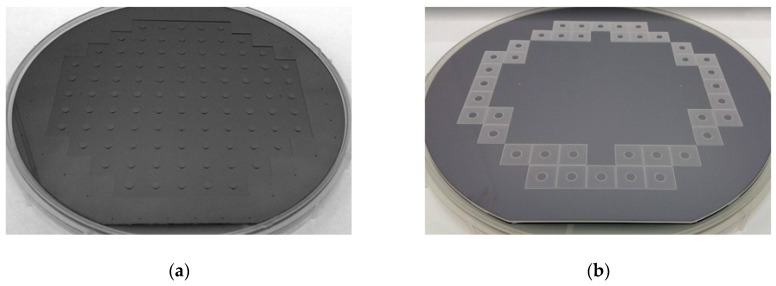
Pillars formed on rear of wafer: (**a**) Type I-A and (**b**) Type II-A.

The pillar formed at the bottom of the wafer causes a vacuum leak, making it impossible for the chuck of the manufacturing equipment to clamp the wafer. In addition, the pillar degrades the backside cooling efficiency of the DRIE process. For these reasons, the pillar is preferably formed at the end of the chip process (just before dicing). The Type I-A device was fabricated following these steps. However, because the fabrication process for the Type II-A device exhibits the low etching selectivity of photoresist to glass, the complexity of the pillar formation process increases [31]. To solve this problem, we formed the pillar first in the manufacturing process of the Type II-A device and then etched the silicon device layer. The vacuum leak problem in the wafer chuck was solved by forming the pillar in an area that did not interfere with the vacuum holes of the wafer chuck. As shown in Figure 12, 97 Type I-A dies were produced on 6-in. wafers, while only 40 Type II-A dies were manufactured on 6-in. wafers.

### 4.5. Structural Reliability of the Pillar Die Attach

The shear strength of the pillar die attach was greater than 35 N. This shear strength value indicates that adequate bonding stability could be achieved for a high-g environment, even with partial die bonding. Additionally, as a result of a modal analysis of the Type I-A and Type II-A with the pillar structure, the frequencies of the first mode were 40.15 kHz and 24.28 kHz, respectively. This result shows that the mechanical resonance problems of the isolator structures using beam elements to reduce packaging stress are unlikely to occur in the pillar structure.

### 4.6. Change in Die Warpage Due to Temperature

The variation in the packaging stress due to changes in the temperature (thermal stress) of each test DRA was evaluated in terms of the change in die warpage. For a dummy die, die warpage is the maximum height difference of the line profile across the die. However, if a device die is used, a continuous line profile across the die cannot be obtained. Thus, in this study, die warpage was defined as the difference between the average height of the four electrode anchors located at the center of the die and the average height of the four stoppers located at the four edges of the die. 

**Figure 13:**
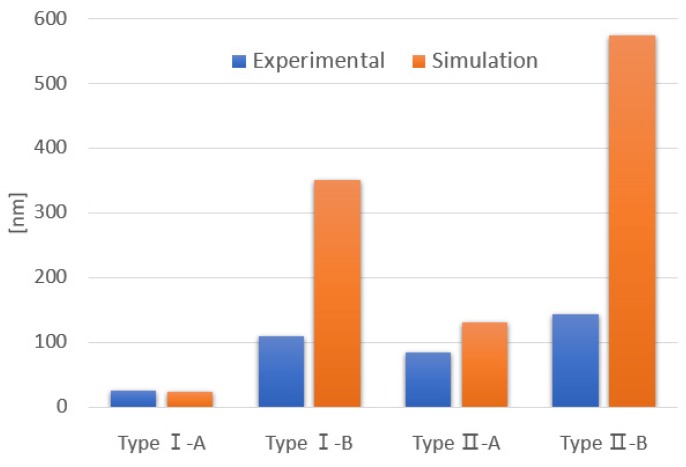
Change in die warpage due to temperature change from 25 °C to 85 °C.

We evaluated the changes in die warpage by conducting a simulation in ANSYS 19.1, a commercial finite element analysis program, and experiments with an optical profiler. The temperature-dependent material properties of the die materials used in the simulation were referenced from the literature [32]. However, the temperature-dependent material properties of the adhesive TB3303N and the ceramic package were not known. Thus, the manufacturer-specified material properties at room temperature were used instead. Figure 13 shows the simulated and experimental results of the change in die warpage obtained by varying the temperature from 25 °C to 85 °C. In the simulation, the thermal stress reduction effect of the pillar die attach compared to the four-dots die attach was 14.6 times and 4.4 times in cases of the Type I and Type II devices, respectively. This showed that the pillar die attach structure formed using the GOS wafers more effectively reduced the thermal stresses than the Type II device formed using the glass wafers. Similar trends were observed in the experimental results. This result can be explained by the change in die warpage due to the CTE differences between the packaging materials.

The multi-layered structure of the test devices could be simplified, except for the adhesive and the thin metal film on the surface of the ceramic package. This is because the considerably thinner materials or those with a lower modulus compared to the other packaging materials, such as the adhesives and the thin metal films, can be estimated as having no significant influence on stress development in the die [33]. In addition, the silicon device layer can be considered to have a negligible influence on die warpage because most of the silicon device layer is a moving structure. With these approximations, the Type I and Type II devices were simplified into glass/Si/ceramic and glass/ceramic multi-layered structures, respectively. As a result, the Type I device with two bonding interfaces of glass/Si and Si/ceramic had a stress compensation effect because the bending directions of the two bonding interfaces due to temperature change were opposite. Furthermore, as shown in Table 4, the CTE difference between silicon and ceramic was about 5.5 times greater than that between glass and silicon. Therefore, the stress compensation was more effective in the pillar die attach, in which the effective bonding area of silicon and ceramic was limited to the pillar area (the effective bonding area of the four-dots die attach was wider than that of the pillar die attach because it included the areas between each of the adhesive dots). The simulation showed that the bending direction of the glass/Si bonding interface changed to the opposite direction at 110 °C. Therefore, the stress compensation based on this structure could be applied to most MEMS devices with a general operating temperature range.

When the change in the die warpage increased, the gap between the simulation and the experimental results widened. This discrepancy can probably be ascribed to the nonlinear and temperature-dependent properties of the TB3303N adhesive. Future studies on the TB3303N adhesive will help reduce the difference in results between the simulations and experiments.

## 5. Conclusions

This study proposed a partial die bonding process that employs a pillar structure to reduce the asymmetric packaging stress and thermal stress in SOG MEMS devices. The pillar structure reduces the asymmetric packing stress by controlling the adhesive spreading area with a fillet that compensates for various error factors in the die attach process. Using a GOS wafer as a handle substrate in the SOG process makes pillar fabrication simpler than that when using a conventional glass wafer, and the packaged devices have a structure in which the thermal stress is compensated for by two bonding interfaces that bend in opposite directions when the temperature changes. The asymmetric packaging stress of the DRA fabricated with the proposed method (Type I-A) was significantly lower and the thermal stress was reduced by 6.38 times compared to that of the DRA fabricated with the conventional method (Type II-B) in an environment with a temperature increase of 60 °C. 

## Figures and Tables

**Table 1:** Definitions of DRA types. GOS: glass-on-silicon.

	Handle Substrate	Die-Attach Scheme
Type I-A	GOS	Pillar
Type I-B	GOS	Four-dot
Type II-A	Glass	Pillar
Type II-B	Glass	Four-dot

**Table 2:** Deformation ratio of both proof masses due to packaging.

	ΔZ_L_ [nm]	ΔZ_R_ [nm]	Deformation Ratio
Type I-A	76	78	1.026
Type I-B	189	331	1.751
Type II-A	102	94	1.085
Type II-B	412	133	3.098

**Table 3:** Output frequency difference in the two DETF resonators.

	f_0L_ [Hz]	f_0R_ [Hz]	Frequency Difference [Hz]
Type I-A	31,021	30,974	47
Type I-B	31,787	31,922	135
Type II-A	30,228	30,283	55
Type II-B	32,212	32,425	213

**Table 4:** Coefficients of thermal expansion of packaging materials at room temperature.

	Glass	Silicon	Ceramic
**CTE** **[ppm/°C]**	3.25	2.60	6.20
